# Beneficial Effects of Apple Vinegar on Hyperglycemia and Hyperlipidemia in Hypercaloric-Fed Rats

**DOI:** 10.1155/2020/9284987

**Published:** 2020-07-10

**Authors:** Driss Ousaaid, Hassan Laaroussi, Meryem Bakour, Asmae ElGhouizi, Abderrazak Aboulghazi, Badiaa Lyoussi, Ilham ElArabi

**Affiliations:** Laboratory of Natural Substances, Pharmacology, Environment, Modeling, Health and Quality of Life (SNAMOPEQ), Faculty of Sciences Dhar El Mahraz, University Sidi Mohamed Ben Abdellah, Fez, Morocco

## Abstract

The main objective of this study is to determine the effects of apple vinegar on the metabolic changes caused by hypercaloric diet in Wistar rats. Apple vinegar was first analyzed to find out the total acidity, the polyphenolic and flavonoid contents, the total antioxidant capacity, and the free radical scavenging activity. *In vivo* study on adult male and female Wistar rats was conducted by administering a drink containing either 10% D-glucose or water (control) for five weeks. Apple vinegar is administered daily by gavage (2 mL/kg) to rats fed D-glucose for 5 weeks. The results showed that the polyphenolic content in apple vinegar was 148.02 ± 10.16 mg GAE/100 mL, flavonoid content was 22.93 ± 0.73 QE/100 mL, and total antioxidant capacity was 13.4 ± 0.47 mg AAE/100 mL. Free radical IC_50_ apple vinegar scavenging activity (DPPH) was 0.74 ± 0.154 *μ*L/mL. The total acidity was (3.24 ± 0.02 mg AAE/100 mL). The treatment during five weeks with D-glucose leads to increased plasma glucose, lipid profile, hepatic enzyme levels, urea, and creatinine. Simultaneous treatment with apple vinegar improves the parameters studied. These results clearly show that the daily consumption of vinegar can reduce the rise in blood sugar and lipid profile induced by hypercaloric diet in rats. Therefore, the use of apple vinegar would have a very beneficial effect in the prevention of metabolic disorders caused by high-caloric food.

## 1. Introduction

Traditionally, the role of diet has been to provide the energy and essential nutrients to support different physiological functions of the body. However, over the years, the role of diet has changed; food is increasingly called upon to provide physiological benefits in terms of management and disease prevention [1]. The changes in lifestyle food (fast food, increased caloric consumption, stored food, and physical inactivity), among others, are key elements facilitating the installation of the metabolic disorders and the onset of many pathologies. Our current feeding is therefore represented by significant carbohydrate contributions. The increase in sugar consumption is related to the accentuation in the prevalence of obesity and insulin resistance predisposing to type 2 diabetes [2].

The hypercaloric diet (HCD) is the principal factor which causes the development of metabolic risk factor of cardiovascular diseases, hypercholesterolemia, hypertension, hyperglycemia, type 2 diabetes, and some cancer types [3–5]. Investigations have shown that chronic glucose feeding induces hypertension, insulin resistance, hyperglycemia, and higher vascular oxidative stress [6–9].

Hyperglycemia is often associated with serious complications such as lipid profile alteration, insulin resistance, liver toxicity, renal dysfunction, retinopathy, and cardiovascular diseases [10, 11]. Effective methods to reduce the onset of diabetes include the control of postprandial hyperglycemia, hyperlipidemia, and the inhibition of lipid and carbohydrate hydrolyzing enzymes [12]. This hyperglycemia represents a key factor for the development of oxidative stress and reactive oxygen species (ROS) [13].

Oxidative stress is at origin of several pathologies such as diabetes. Secondary metabolites contained in natural products such as apple vinegar would be a powerful antioxidant to prevent oxidative stress [14].

There is a need to find alternative solutions to reduce the risk, spread, and progression of metabolic diseases. Several studies have been focused on the identification of alternative therapies to decrease disease incidence, in particular disaccharidase inhibitors and alpha-glucosidase inhibitor [15, 16].

Apple vinegar is widely used and appreciated by the Moroccan population and around the world. Several studies clearly demonstrated many benefits of vinegar consumption such as glucose-lowering effect in patient with glucose abnormalities [16–18], improved insulin sensitivity in insulin-resistant patients [19], decreasing the glycemic index of carbohydrate food for people with and without diabetes [19], antihyperlipidemic [18], hepatoprotective effect [20, 21], and modulation of lipid peroxidation [22].

Since there has been no study on the therapeutic effect of the apple vinegar on glycemic induced by a high carbohydrate diet, this work was conducted to determine whether a subchronic treatment during five weeks with apple vinegar would have a potential effect with regard to the modulation of hyperglycemia and hyperlipidemia in HCD-fed rats.

## 2. Materials and Methods

### 2.1. Vinegar Sample

Apple vinegar was purchased from a local cooperative in Midelt area (32° 41′ 06.7^″^ N 4° 44′ 42.4^″^ W). The raw material used to produce this vinegar is made from two varieties of apple (Golden delicious and Starking delicious). The sample was kept in the fridge (3°C) until it was used for the various experiments carried out in this work.

### 2.2. The Antioxidant Contents and Activities of Apple Vinegar

The total polyphenolic content in the apple vinegar sample was determined by Folin-Ciocalteu reagent using a method described by Singleton et al. [23]; the value of total polyphenolic compounds was expressed as milligrams of gallic acid equivalent per 100 mL of vinegar. Total flavonoid content was determined using the method described by Kong et al. [24]; the outcome was expressed as milligrams of quercetin equivalent per 100 mL of vinegar.

The total antioxidant capacity of apple vinegar was examined using the method reported by Zengin et al. [25]. The result was expressed as milligrams of ascorbic acid equivalent per 100 mL of vinegar. The scavenging activity of apple vinegar for the radical 2,2-diphenyl-1-picrylhydrazyl (DPPH) was measured as described by Miguel et al. and Laaroussi et al. [26, 27]. The scavenging activity was estimated based on the percentage of DPPH radical scavenged using the following equation:

(1) $$\begin{array}{c} \%\text{Inhibition}=\left(A_{0}-A_{1}/A_{0}\times 100\right. \end{array}$$

*A*_0_ is the absorbance of the control; *A*_1_ is the absorbance of the sample.

The IC_50_ DPPH was calculated from the obtained graph of inhibition percentage of radical DPPH.

### 2.3. Total Acidity of Apple Vinegar

The total acidity was determined by titration according to the French standard [28].

### 2.4. Animals and Procedures

Adult male and female rats weighing between 168.5 ± 8.5 g and 132 ± 8 g, respectively, were obtained from animal house breeding center, Faculty of Sciences, Dhar Al-Mahraz Fez, and were housed under normal environmental conditions (25 ± 1°C) (55 ± 5% humidity on a 12-hour light-dark cycle). The care and handling of the animals were in accordance with the internationally accepted standard guidelines for the use of animals, and the protocol was approved by the institutional committee on animal care following the French Technical Specifications for the Production, Care and Use of the Laboratory Animals.

The rats were randomly allocated into three groups of eight rats each (4 females, 4 males) treated for 5 weeks as follows: group 1: represents the control group, had free access to tap water only and normal diet; group 2: had free access to drinking solution of 10% D-glucose and to a normal diet; and group 3: had free access to 10% D-glucose and treated daily by gavage with apple vinegar (2 mL/kg). The body weight was measured in the first and the last day of treatment.

After 5 weeks of treatment, the rats were fasted overnight (16 h) and sacrificed by decapitation after light ethyl urethane anesthesia. Blood was withdrawn from each rat, and plasma was recovered for various biochemical determinations.

### 2.5. Biochemical Methods

The plasma was immediately separated by low-speed centrifugation at 1500 × g for 15 min. Plasma was obtained to analyze blood glucose, total cholesterol (TC), triglycerides (TG), and high-density lipoprotein cholesterol (HDL-C) and LDL cholesterol (LDL-C), total protein, alanine aminotransferase (ALT), aspartate aminotransferase (AST), lactate dehydrogenase (LDH), serum creatinine, urea, calcium (Ca^2+^), sodium (Na^+^), potassium (K^+^), and chloride (Cl^−^).

### 2.6. Statistical Analysis

The data were expressed asmean ± SDvariable reading in each group. Statistical comparisons between the groups were performed with one-way analysis of variance (ANOVA) followed by Dunnett test to compare all columns with control column (GraphPad Prism 5 software). The data followed normal distribution.

## 3. Results

### 3.1. Total Polyphenolic Content (TPC), Total Flavonoid Content (TFC), Antioxidant Activities (DPPH and TAC), and Total Acidity (TA) of Apple Vinegar

TPC, TFC, TA, TAC, and DPPH (IC_50_) values of apple vinegar are shown in Table 1. Generally, the total polyphenolic content was 148.02 ± 10.16 mg GAE/100 mL, flavonoids was 22.93 ± 0.73 QE/100 mL, and total antioxidant activity was 13.4 ± 0.47 mg AAE/100 mL. The IC_50_ of free radical scavenging activity of apple vinegar (DPPH) was (0.74 ± 0.154 *μ*L/mL). The total acidity was demonstrated by milligrams of acetic acid equivalent per 100 mL of vinegar (3.24 ± 0.02 mg AAE/100 mL).

### 3.2. Biological Assessments

#### 3.2.1. Effect of D-Glucose and Apple Vinegar on Body Weight and Body Weight Gain

Table 2 resumes the change in body weight of rats in the experimental groups. The body weight gain was not significantly modified either by D-glucose feeding or by apple vinegar despite a trend to a diminution of body weight gain in both sexes (males and females), which was observed with apple vinegar treatment (group 3).

#### 3.2.2. Effect of D-Glucose and Apple Vinegar on Glycemia

**Figure 1:**
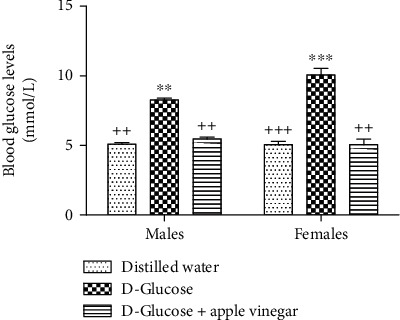
Effect of D-glucose and apple vinegar on the blood glucose levels in experimental animals. ^∗^*p* < 0.05 vs. the distilled water group. ^+^*p* < 0.05 vs. the D-glucose group.

Figure 1 shows that five weeks of treatment with D-glucose caused a significant increase in blood glucose level (*p* < 0.05) (8.27 ± 0.12 mmol/L and 10.06 ± 0.46 mmol/L in male and female rats, respectively) in comparison to control (5.08 ± 0.11 mmol/L and 5.06 ± 0.23 mmol/L in males and females, respectively) and rats treated by D-glucose combined with apple vinegar (5.09 ± 0.42 mmol/L and 5.44 ± 0.15 mmol/L for male and female rats, respectively).

#### 3.2.3. Effect of D-Glucose and Apple Vinegar on Plasma Lipid Profile

Table 3 shows concentrations of the serum which was collected in the three groups of rats of our experiment, in total cholesterol (TC), in triglycerides (TG), in low-density lipoproteins (LDL-C), or in high-density lipoproteins (HDL-C).

In group 2 which underwent a subchronic D-glucose supply, the LDL-C concentration increased significantly in rats of both sexes, the TC increased significantly in female rats, while no significant increase was observed in TG levels. On the other hand, the HDL-C concentration decreased significantly (*p* < 0.05) in rats of both sexes. In group 3, apple vinegar supplemented with D-glucose made it possible to reduce the serum concentrations of LDL-C, TG, and TC but not statistically significant.

#### 3.2.4. Effect of D-Glucose and Apple Vinegar on Hepatic Enzymes

The effect of D-glucose intake and apple vinegar (AV) coadministration on hepatic enzymes in different groups is summarized in Table 3. D-Glucose increased significantly the levels of plasma aspartate aminotransferase (AST) (*p* < 0.05) and LDH (*p* < 0.05) in both sexes but not significantly increased ALT of both sexes compared with the other groups. The levels of plasma urea were found to be significantly (*p* < 0.05) increased but not significantly concerning creatinine in the D-glucose-fed group when compared to the control group and D-glucose combined with apple vinegar-treated group.

#### 3.2.5. Effect of D-Glucose and Apple Vinegar on Kidney Indices of Toxicity

In group 2 of rats fed D-glucose for 5 weeks, there are no significant changes in the plasma total protein concentrations but there is a slight increase in creatinine levels compared to the control group; on the other hand, there is a significant increase in urea levels. The addition of apple vinegar to group 3 causes a significant decrease in urea levels and a slight decrease in creatinine levels. We also note, in the same group, a certain increase in the levels of total proteins (Table 3).

#### 3.2.6. Effect of D-Glucose and Apple Vinegar on Plasma Electrolytes

In an attempt to evaluate the effect of subchronic D-glucose administration and apple vinegar supplementation on plasma electrolytes, we have measured the plasma levels of calcium (Ca^2+^), sodium (Na^+^), potassium (K^+^), and chloride (Cl^−^) in rats of different groups. Results are summarized in Table 3. It was clearly shown that the plasma sodium and chloride levels were not significantly changed either by D-glucose feeding alone or combined with apple vinegar in comparison with the control group. The plasma potassium levels in both sexes of group 2 were not changed significantly. The coadministration of D-glucose and apple vinegar (AV) decreased significantly (*p* < 0.05) the plasma potassium levels in rats of both sexes. Concerning plasmatic calcium levels, the D-glucose intake (10% in water) for 5 weeks as well as the simultaneous administration of D-glucose and apple vinegar (2 mL/kg) increased significantly (*p* < 0.05) the calcium levels as compared to the nondiabetic group in male and female rats (group 1). In addition, a significant difference was observed between male rats of group 3 and both sexes of group 2.

## 4. Discussion

Apple vinegar is very well known for its unsuspected health benefits; it is rich in bioactive molecules such as polyphenolic compounds known for its several therapeutic effects [29]. The current study showed that five-week treatment with D-glucose increased glycemia, hepatic enzymes, and lipid profile levels; these results are in agreement with previous studies [9, 30, 31]. Furthermore, subchronic feeding with D-glucose combined with apple vinegar modulates the different studied parameters. Importantly, until now, there are no studies that have been conducted to determine the efficacy of apple vinegar on hyperglycemia and hyperlipidemia caused by D-glucose feeding in rats of both sexes.

Decreasing postprandial hyperglycemia is a therapeutic way, which delays glucose absorption [32]. Previously, there are many authors who reported that the apple vinegar can play an important role in food digestion. Additionally, in prophetic medicine, the Prophet Mohammed peace be upon him recommended drinking vinegar in the prophetic hadeeth: “vinegar is the best edible.” Moreover, the apple vinegar has been shown to reduce fasting blood glucose [16, 19, 33–35], by reducing the post-prandial insulin response [35]. The intake of apple vinegar ameliorates the insulin sensitivity and increase the uptake of glucose in the skeletal muscles [17]. Acetic acid (a major component of vinegar) was suggested as a key to slowing down of gastric emptying [34] and decreasing of disaccharidase activity in the small intestinal [15], intestine maltase, lactase, and sucrose activities [18]. Additionally, it promotes the uptake of glucose by muscle performance [17, 36], consequently, decreasing fasting blood glucose.

The finding results revealed that apple vinegar was able to reduce moderately the concentration of total cholesterol (TC) in female rats as well as triglycerides and low-density lipoprotein cholesterol (LDL-C) in rats of both sexes. In opposite, it increased the level of high-density lipoprotein cholesterol (HDL-C) in male and female rats.

Data are in agreement with previous studies which have shown that the apple vinegar modulates the lipid profile [37, 38]. The regulation of lipids has an effect on weight loss; nowadays, many studies have shown that the apple vinegar promotes antiobesity effects [22] and exerts its beneficial effects via scavenging free radicals [22, 39]. Our data showed that the treatment with apple vinegar modulates the weight gain in both sexes compared with group of rats treated with D-glucose.

In the present study, the supplementation of apple vinegar to the HCD decreases the hepatic enzyme levels. It could be due to their high content of bioactive molecules such as polyphenolic compounds [39].

The results of antioxidant contents obtained in apple vinegar are in accordance with those reported by previous reports [39, 40]. The richness of vinegar in bioactive molecules such as polyphenols and flavonoids depends mainly on raw matter [41].

Regarding the liver function, the subchronic administration of D-glucose induced a high increase of plasmatic AST and LDH levels in male and female rats which are in accordance with the findings of Goboza et al. [42]. The increase of plasma hepatic enzymes is a major index of liver damage and hepatic cell necrosis which causes molecular destabilization of membrane cell phospholipids and thus the leakage of cytoplasmic enzymes [43]. It was found that HCD induces metabolic disorders and oxidative stress with serious hepatocyte injury surpassing antioxidant defense systems [40].

Oxidative stress is the main cause of many liver problems; therefore, the attenuation of reactive oxygen species (ROS) caused by HCD is the effective approach to prevent the extent of liver damage. Indeed, the simultaneous administration of D-glucose and apple vinegar (group 3) for five weeks reduced significantly the plasma levels of AST and LDH as compared to nontreated diabetic rats (group 2). These results were in agreement with previous studies [16, 20]. Furthermore, our data indicate that D-glucose administrated alone has no significant effect on the normal serum levels of ALT. The protective effect of apple vinegar might be due to its richness in various chemical components and bioactive molecules [44].

In addition, the administration of apple vinegar has been reported to decrease lipid peroxidation in ovariectomized mice, promotes GSH-Px activity which can prevent oxidative stress [22], and promotes the enzymatic antioxidant defense systems [21].

The evaluation of plasma electrolytes such as (Ca^+^, Na^+^, and K^+^) is a crucial step in the diagnosis of metabolic disorders, in particular type 2 diabetes (T2D), because they play a key role in the regulation of blood pressure [45]. Sodium is an electrolyte mainly involved in the development of high blood pressure (hypertension) and other cardiovascular complications. The hypokalemia expressed by diabetic rats cotreated with apple vinegar (group 3) may be due to a compensatory response from the renal system to moderate the concentration of Na ^+^ and thus maintain the balance of blood sodium [46].

Furthermore, the present study revealed a significant increase in plasma calcium concentrations in nontreated diabetic rats as compared to the control group. In fact, numerous studies have been reported that the high plasma calcium level was directly associated with T2D risk [47, 48]. However, the daily coadministration of apple vinegar (2 mL/kg) was not able to ameliorate the calcium plasma level. That is probably related to its high concentration in macroelements, especially calcium. In addition, it has a stimulating effect on calcium absorption [49, 50]. This may improve the reduction of blood pressure by the renin-angiotensin system through the inhibition of renin release [50].

Creatinine is a result of the breakdown of muscle creatine, while urea is a metabolic waste of proteins [51]; the increase of these two renal biomarkers in nontreated diabetic group is a sign of nephropathy diabetic. Weekers and Krzesinski showed that the diabetic nephropathy is due to an alteration of the renal glomeruli owing to glycotoxicity, oxidative stress, and high intraglomerular pressure [52].

In this study, the oral administration of apple vinegar at a dose of 2 mL/kg decreased urea levels in both sexes and creatinine in female rats but not significantly. This effect could be due to its phenolic components particularly pyrogallol and catechin [38]. Previous studies reported that catechin and pyrogallol can prevent kidney damage and lower the levels of creatinemia and uricemia [18, 53].

## 5. Conclusion

The chemical analysis of apple vinegar revealed the presence of bioactive compounds such as a high content of flavonoids which might be responsible for the exceptional biological properties of apple vinegar in this case the antioxidant activity, the antihyperglycemic activity, and the antihyperlipidemic activity.

Our results show that the hypercaloric diet (D-glucose) is associated with increase in blood sugar, triglycerides, cholesterol, LDL, liver enzyme levels, urea, and creatinine.

The daily intake of vinegar could offer promising protective effects on metabolic changes induced by HCD.

## Figures and Tables

**Table 1:** Total acidity, antioxidant contents, and antioxidant activities of apple vinegar.

	TPC (mg GAE/100 mL)	TFC (mg QE/100 mL)	TAC (mg AAE/100 mL)	IC_50_DPPH (*μ*L/mL)	TA (mg AAE/100 mL)
Apple vinegar	148.02 ± 10.16	22.93 ± 0.73	13.4 ± 0.47	0.74 ± 0.154	3.24 ± 0.02

**Table 2:** Effect of D-glucose and apple vinegar on body weight and body weight gain.

	Body weight (g)				Body weight gain (g)	
	Initial		Final			
Groups	Males	Females	Males	Females	Males	Females
Group 1	175.5 ± 2.12	126 ± 2.82	195.5 ± 3.53	141.5 ± 0.70	20 ± 5.65	15.5 ± 2.12
Group 2	168.5 ± 2.12	128 ± 5.65	200.5 ± 10.60	147.5 ± 17.67	32 ± 8.48	19.5 ± 12.02
Group 3	172 ± 2.82	126 ± 2.82	185.5 ± 6.36	138 ± 5.65	13.5 ± 3.53	12 ± 8.48

**Table 3:** Effect of D-glucose and apple vinegar on plasma lipid profile, liver function, kidney indices of toxicity, and plasma mineral element status.

Biochemical parameters	Group 1		Group 2		Group 3	
	Males	Females	Males	Females	Males	Females
Plasma lipid profile						
TC (mg/dL)	42.5 ± 3.53	44 ± 1.41	55 ± 14.14	73.5 ± 2.12^∗^	60 ± 5.65	60 ± 4.24
TG (mg/dL)	54.5 ± 13.43	43.5 ± 2.12	73 ± 2.82	60 ± 15.55	48 ± 1.41	36 ± 11.31
HDL-c (mg/dL)	0.24 ± 0.4	0.21 ± 0.15	0.14 ± 0.01^∗^	0.14 ± 0.05^∗^	0.21 ± 0.01	0.15 ± 0.005
LDL-c (mg/dl)	42.5 ± 0.70	44.5 ± 2.12	55.5 ± 4.94^∗^	52.5 ± 2.12^∗^	47 ± 1.41	46.5 ± 2.12
Liver function						
AST (U/L)	109.5 ± 3.53^+^	119 ± 1.41	159.5 ± 7.77^∗^	174 ± 15.55^∗^	93.5 ± 7.77^+^	129 ± 12.79
ALT (U/L)	35 ± 2.82	20.5 ± 10.60	42.5 ± 3.53	50 ± 2.82	17.5 ± 2.12^∗^^+^	20 ± 9.89
LDH (U/L)	396.5 ± 10.60^+^	472 ± 84.85^+^	852.5 ± 24.93^∗^	665.5 ± 62.93^∗^	373.5 ± 38.89^+^	405 ± 17.9^+^
Kidney indices of toxicity						
Total proteins (mg/dL)	56.5 ± 12.02	49.5 ± 3.53	51.5 ± 2.12	52.5 ± 2.12	60 ± 5.65	60 ± 4.24
Urea (mmol/L)	0.31 ± 0.01^++^	0.37 ± 0.25^+^	0.39 ± 0.4^∗∗^	0.41 ± 0.15^∗^	0.25 ± 0.9^∗^^+^	0.27 ± 0.13^∗^^+^
Creatinine (*μ*mol/L)	5 ± 1.41	4.5 ± 0.5	6.5 ± 0.5	7 ± 1	6.5 ± 1.5	5.5 ± 0.5
Plasma mineral element status						
Ca^2+^ (mmol/L)	20.5 ± 0.70^+^	19 ± 1.41^+^	64 ± 1.41^∗^	86.5 ± 9.19^∗^	96.5 ± 2.10^∗^^+^	82.5 ± 10.60^∗^
Na^2+^ (mmol/L)	140.5 ± 0.70	139.5 ± 0.70	128.5 ± 14.84	137.5 ± 2.13	141.5 ± 0.70	139 ± 0.1
K^+^ (mmol/L)	5.25 ± 0.05	5.05 ± 0.025	6.2 ± 0.3	5.7 ± 0.1	4.2 ± 0.1^∗^^+^	4.7 ± 0.2^+^
Cl^−^ (mmol/L)	97 ± 2.82	101.5 ± 0.71	88.5 ± 2.12	106.5 ± 2.11	102.5 ± 0.69	102 ± 4.24

^∗^ *p* < 0.05 vs. group 1. ^+^*p* < 0.05 vs. group 2.

## Data Availability

The data used to support the findings of this study are available from the corresponding author upon request.
